# Relationship between Localization on Cellular Membranes and Cytotoxicity of *Vibrio vulnificus* Hemolysin

**DOI:** 10.1371/journal.pone.0026018

**Published:** 2011-10-20

**Authors:** Hiroyuki Sugiyama, Takashige Kashimoto, Shunji Ueno, Hayato Ehara, Toshio Kodama, Tetsuya Iida, Nobuyuki Susa

**Affiliations:** 1 Laboratory of Veterinary Public Health, School of Veterinary Medicine, Kitasato University, Towada, Aomori, Japan; 2 Department of Bacterial Infections, Research Institute for Microbial Diseases, Osaka University, Suita, Osaka, Japan; 3 International Research Center for Infectious Diseases, Research Institute for Microbial Diseases, Osaka University, Suita, Osaka, Japan; Bernhard Nocht Institute for Tropical Medicine, Germany

## Abstract

*Vibrio vulnificus* secretes a hemolysin/cytolysin (VVH) that induces cytolysis in target cells. A detergent resistant membrane domain (DRM) fraction of the cells after sucrose gradient centrifugation includes cholesterol-rich membrane microdomains which have been called “lipid rafts”. It was reported that some pore-forming toxins require association with DRM and/or lipid rafts to exert their cytotoxicity. It has also been thought that cellular cholesterol is involved in VVH cytotoxicity because VVH cytotoxicity was inhibited by pre-incubation with cholesterol. However, both cellular localization and mode of action of VVH cytotoxicity remain unclear. In this study, we investigated the relationship between VVH localization on the cellular membrane and its cytotoxicity. Oligomers of VVH were detected from DRM fractions by sucrose gradient ultracentrifugation but all of these oligomers shifted from DRM fractions to non-DRM fractions after treatment with methyl-beta-cyclodextrin (MβCD), a cholesterol sequestering agent. On the other hand, immunofluorescence analysis showed that VVH did not co-localize with major lipid raft markers on cellular membrane of CHO cells. These data suggested that VVH localized at membrane regions which are relatively abundant in cholesterol but which are not identical with lipid rafts. To determine the linkage between localization and cytotoxicity of VVH, cytotoxicity was evaluated in MβCD-treated CHO cells. The cytotoxicity of VVH was not decreased by the MβCD treatment. In addition, the amount of VVH oligomer did not decrease in MβCD-treated CHO cells. Thus, we found that the amount of oligomer on cellular membrane is important for induction of cytotoxicity, whereas localization to lipid rafts on the cellular membrane was not essential to cytotoxicity.

## Introduction

*Vibrio vulnificus* is an opportunistic pathogen that results in a high mortality rate (>50%) in septicemia [1]. Primary septicemia in *V. vulnificus* infection is caused by the ingestion of contaminated seafood or through wound infection resulting from exposure to contaminated seawater or marine products [2], [3]. *V. vulnificus* secretes a pore-forming toxin called *Vibrio vulnificus* hemolysin/cytolysin (VVH) that is a possible virulence factor [4], [5]. Most studies of the cellular intoxication of VVH have focused on the hemolytic mechanism. VVH monomer binds to cell membrane to form SDS-resistant oligomers [6]. These oligomers form small ion-permeable pores that induce hemolysis via colloid osmotic shock [7]. Cholesterol neutralizes the hemolytic activity of VVH in a concentration-dependent manner, and the VVH monomer was converted into an oligomer by mixing with cholesterol [8]. Therefore, cholesterol has been thought to be one of the cellular receptors for VVH.

On cellular membranes, there are several microdomains termed lipid rafts that are characteristically rich in cholesterol, sphingolipid, glycosylphosphatidylinositol (GPI)-anchored proteins, Fas/CD95, Src kinases, small G proteins, and heterotrimeric G proteins. These elements are thought to serve as platforms for the assembly of signaling complexes [9], [10]. In addition, lipid rafts are important for bacteria or viruses to penetrate to host cells [11], [12], [13]. Lipid rafts are detected as detergent resistant membranes (DRMs) by sucrose gradient ultracentrifugation, and DRMs are characterized biochemically by their resistance to detergents, such as Triton X-100, at low temperature [14], [15]. Until recently, it had been thought that DRMs and lipid rafts were the same. However, it is now thought that DRMs are similar to lipid rafts, but not identical. Because addition of Triton X-100 may induce not only enhancement of liquid-ordered domain formation but also fusion of existing lipid rafts, this treatment forms some large confluent membrane aggregates in the cells [16], [17]. Although analysis using sucrose gradient ultracentrifugation is still controversial because of the issues mentioned above, this method using detergent remains in general use for separation of lipid rafts in cell membranes.

Recently, it was also suggested that lipid rafts could be classified by their associated molecules. Shogomori et al. reported that sphingomyelin-rich domains are distinct from GM1-rich domains [18]. Fujita et al. reported GM3-rich domains did not co-exist with GM1-rich domains [19]. Moreover, Matsuda et al. reported that the localization of *Vibrio parahaemolyticus* thermostable direct hemolysin (TDH) was shifted from DRM fractions to non-DRM fractions by MβCD treatment, and that the cytotoxicity of TDH to HeLa cells was decreased by this treatment [20]. On the other hand, the localization and cytotoxicity of aerolysin, a pore-forming toxin produced by *Aeromonas hydrophila*, were not affected by the treatment with MβCD [21], [22]. Thus, localization of pore-forming toxins in their specific-DRMs might be important for these toxins to exert cytotoxicity. However, to date, the localization of VVH in target cell membrane has not been elucidated. In this study, we investigated the linkage between localization and cytotoxicity of VVH. We found that the VVH cytotoxicity was not affected by MβCD-treatment in CHO cells, despite the fact that the VVH fractions were shifted from the DRMs to non-DRMs in the cellular membrane.

## Results

### VVH associates with DRMs

**Figure 1:**
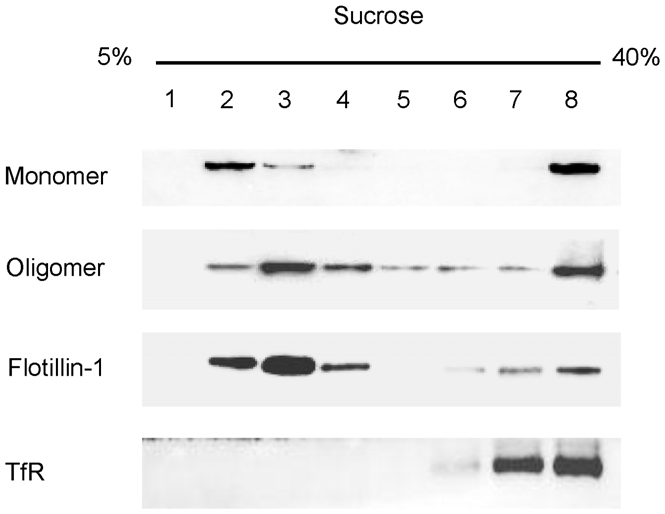
VVH associates with DRMs. CHO cells were incubated with 5 µg/ml VVH at 37°C for 15 min. After being fractionated by sucrose gradient ultracentrifugation, VVH, flotillin-1 (flt-1), and transferrin receptor (TfR) were detected by western blotting using specific antibodies.

VVH may be localized at DRMs, cholesterol-rich microdomains, because cholesterol is thought to be a cellular receptor for VVH. However, binding and association of VVH on cellular membranes remains unclear. To investigate the localization of VVH on cellular membranes, VVH-treated CHO cells were lysed with 1% Triton X-100 and lysate was fractionated by sucrose gradient ultracentrifugation. VVH monomers and oligomers were detected in both DRM and non-DRM fractions with Flotillin-1 or TfR, which are known as major markers of lipid rafts or non-lipid rafts, respectively (Fig. 1). It is known that VVH binds to cellular membrane as a monomer and then forms oligomers by membrane fluidity [8]. Our result indicates that monomers of VVH bind to membrane regions in both DRM and non-DRM fractions, and that these monomers then oligomerize in both regions.

### MβCD changes the localization of VVH

**Figure 2:**
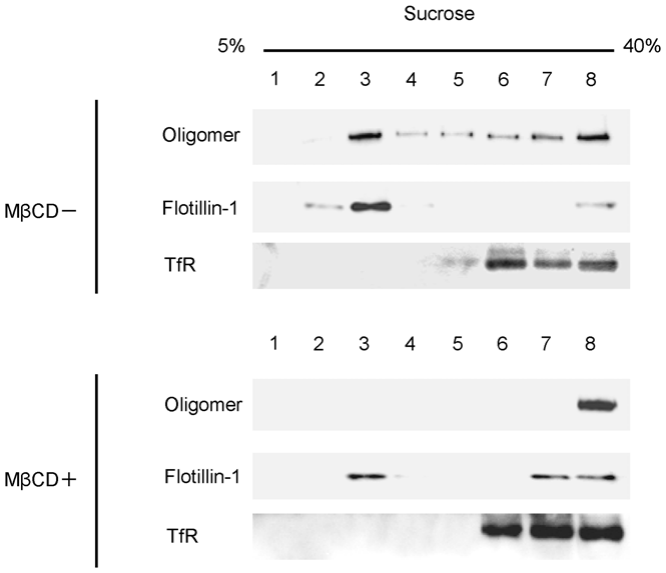
MβCD changes the localization of VVH. CHO cells were incubated with (+) or without (−) 8 mM MβCD at 37°C for 1 h. After incubation, the cells were washed twice with DMEM and incubated with 5 µg/ml VVH at 37°C for 15 min. Cells were lysed, and fractionated. VVH, flotillin-1 (flt-1), and transferrin receptor (TfR) were detected by western blotting using specific antibodies.

We investigated whether MβCD changes the localization of VVH or not. Oligomers of VVH are simultaneously associated with Flotillin-1 and TfR in MβCD untreated cells. However, after treatment with 8 mM MβCD to sequester cellular cholesterol, the oligomer was not found in DRM fractions and was detected only in non-DRM fractions (Fig. 2). These results indicated that the sequestering of cholesterol changes the cellular localization of VVH in CHO cells.

### Localization and cytotoxicity of VVH are not affected by SMase

**Figure 3:**
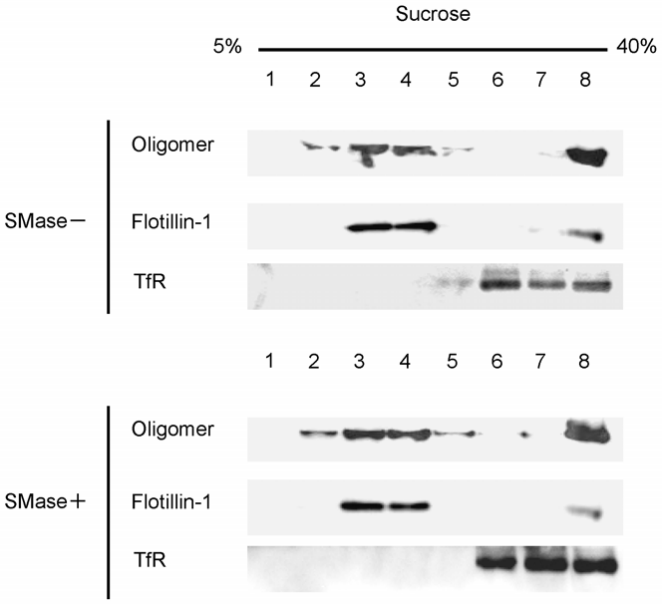
Localization and cytotoxicity of VVH are not affected by SMase. CHO cells were incubated with (+) or without (−) 100 mU/ml SMase at 37°C for 1 h. After incubation, the cells were washed twice with DMEM and incubated with 5 µg/ml VVH at 37°C for 15 min. Cells were lysed, and fractionated. VVH, flotillin-1 (flt-1), and transferrin receptor (TfR) were detected by western blotting using specific antibodies.

It is well known that sphingomyelin is one of the major components of lipid rafts [23]. Therefore, we investigated whether or not the localization of VVH is affected by SMase, which hydrolyzes sphingomyelin into ceramide and phosphorylcholine. Unlike the case with MβCD, localization of VVH was unaffected by SMase (Fig. 3). We used lysenin, which binds sphingomyelin specifically and induces cytotoxicity, as a control for assessment of effect of SMase. VVH could induce cytotoxicity in 100 mU/ml SMase-treated cells (data not shown), whereas the percentage of LDH release by lysenin was decreased from 76.6±4.1% to 0.26±0. 4% in 100 mU/ml SMase-treated cells (data not shown). It was apparent that the localization and cytotoxicity of VVH was unaffected by treatment with SMase.

### VVH does not co-localize with major lipid raft molecules or a non-lipid raft molecule on cellular membrane

**Figure 4:**
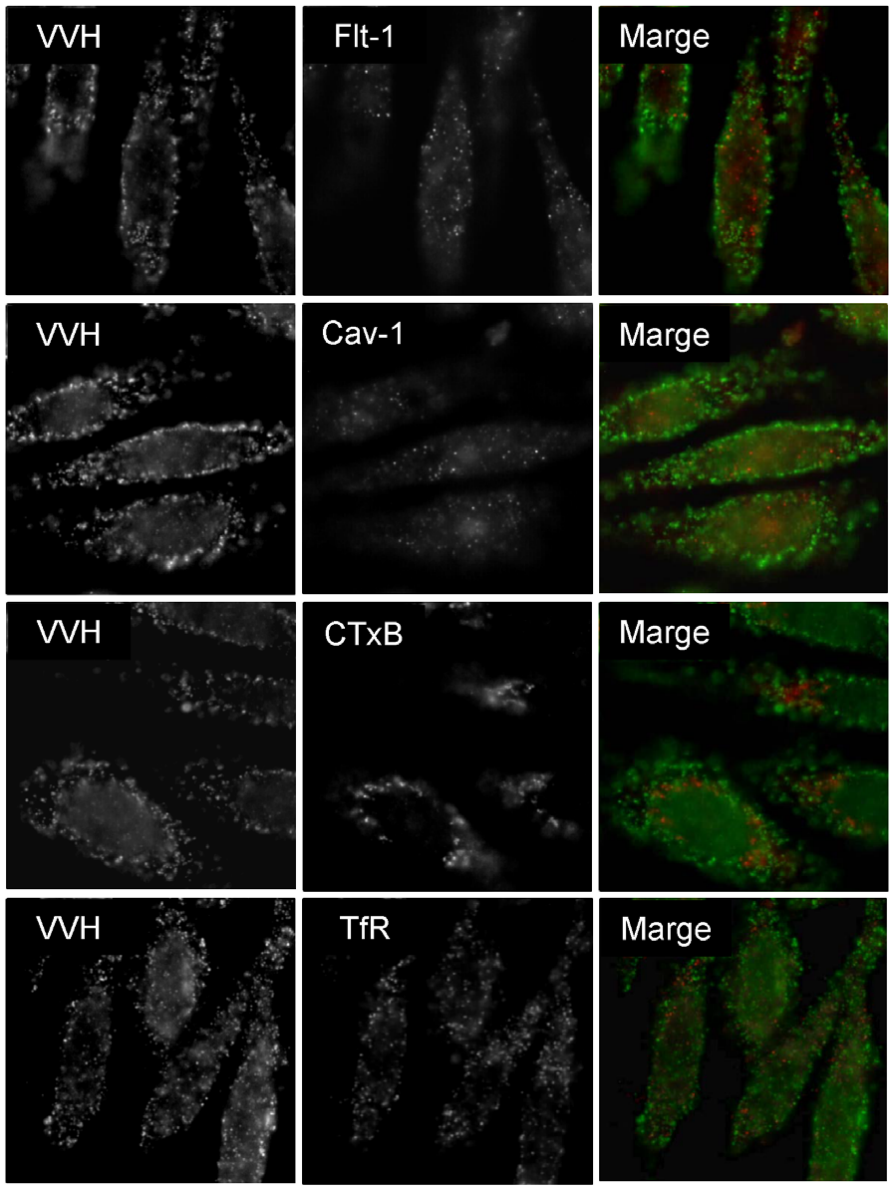
VVH does not co-localize with three major lipid raft molecules or a non-lipid raft molecule. CHO cells were fixed, and then incubated with 5 µg/ml VVH. After washing the cells with PBS, the cells were incubated with anti-VVH and biotin- conjugated CTxB, anti-cav-1, anti-flt-1 or anti-TfR. The cells were probed with Alexa 488-conjugated anti-rabbit anti-body (for VVH) and Alexa 546-conjugated streptavidin (for CTxB) or Alexa 546-conjugated anti-mouse anti-body (for cav-1 and flt-1). Images were obtained by FW4000 fluorescent microscopy.

As shown in Fig. 2, VVH localization was redistributed in MβCD-treated cells, but was not affected by SMase-treatment (Fig. 3). In addition, many recent reports have shown that DRM fractions separated by sucrose gradient ultracentrifugation are not identical to lipid rafts [16], [17]. To assess whether VVH localized at lipid rafts on cellular membrane or not, the localization of VVH on cellular membrane was investigated by immunofluorescence analysis using flt-1, caveolin-1 (cav-1), or cholera toxin subunit B (CTxB), which are known as major lipid raft marker molecules, or TfR as a non-lipid raft marker molecule as the control. VVH did not co-localize with any of these molecules (Fig. 4). These data suggested that VVH does not localize at any of the lipid or non-lipid rafts detected by the marker molecules used in this study. VVH apparently localizes at unique regions where these major lipid raft and non-raft markers are not found.

### Cholesterol sequestering did not affect VVH cytotoxicity in most cell lines

**Figure 5:**
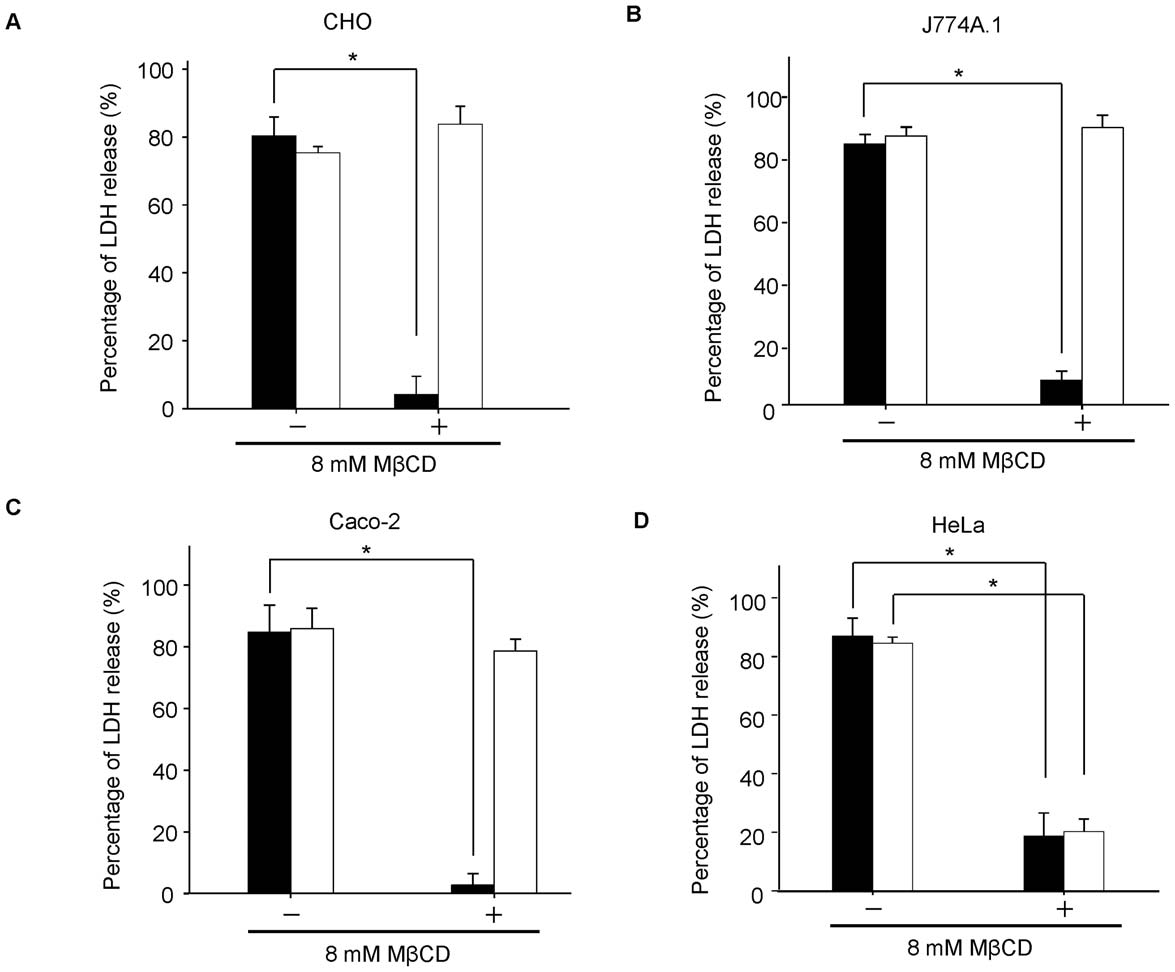
Cholesterol sequestering did not affect VVH cytotoxicity in most cell lines. CHO (A), J774A.1 (B), Caco-2 (C) and HeLa cells (D) were incubated with (+) or without (−) 8 mM MβCD at 37°C for 1 h. After washing the cells, the cells were incubated with 1 µg/ml of VVH (open bar) for 3 h or 50 HU/ml of SLO (closed bar) for 1.5 h at 37°C. The cytotoxicity in these cells was assayed by the release of lactate dehydrogenase (LDH). Data are represented as the mean ± SD and represent more than three independent experiments, each in triplicate wells. *, analysis of variance and Tukey's test, *P*<0.01.

The VVH did not localize at lipid rafts, which are cholesterol rich membrane domains (Fig. 4). On the other hand, it was reported previously that cholesterol sequestering could inhibit the cytotoxicity of VVH in HeLa cells and HL-60 cells [24], [25]. Therefore, cholesterol is thought to be a cellular receptor for VVH. We investigated the effect of cholesterol sequestering on VVH cytotoxicity using the LDH release assay in various cell lines including HeLa cells. LDH is an enzyme confined to the cytoplasm, and its extracellular presence reflects cell damage. SLO, a well known cholesterol dependent cytolysin (CDC), was used in this assay as a control [26], [27]. As shown in Figure 5A, B, and C, 8 mM MβCD has no effect on the percentage of LDH release by VVH, whereas that by SLO was decreased significantly in CHO cells (a Chinese hamster ovary cell line), J774A.1 cells (a mouse reticulum cell sarcoma cell line), and Caco-2 cells (a human colorectal adenocarcinoma cell line). These data indicate that cholesterol sequestering did not affect VVH cytotoxicity in most cell lines. In addition, the cytotoxicity of VVH was not affected by MβCD in CHO cells (Fig. 5A), despite the fact that the distribution of oligomers shifted from DRM fractions to non-DRM fractions in 8 mM MβCD-treated CHO cells (Fig. 2). These results indicate that the localization of VVH at DRMs is not always necessary to exert its cytotoxicity. On the other hand, in HeLa cells (a human cervical adenocarcinoma cell line), the sequestering of cholesterol decreased the amount of LDH release by SLO and VVH only in the 8 mM MβCD-treated cells (Fig. 5D). These data suggest that effect of cholesterol sequestering on VVH cytotoxicity varies by cell line.

### HeLa cells are highly susceptible to sequestration of cholesterol by MβCD

**Figure 6:**
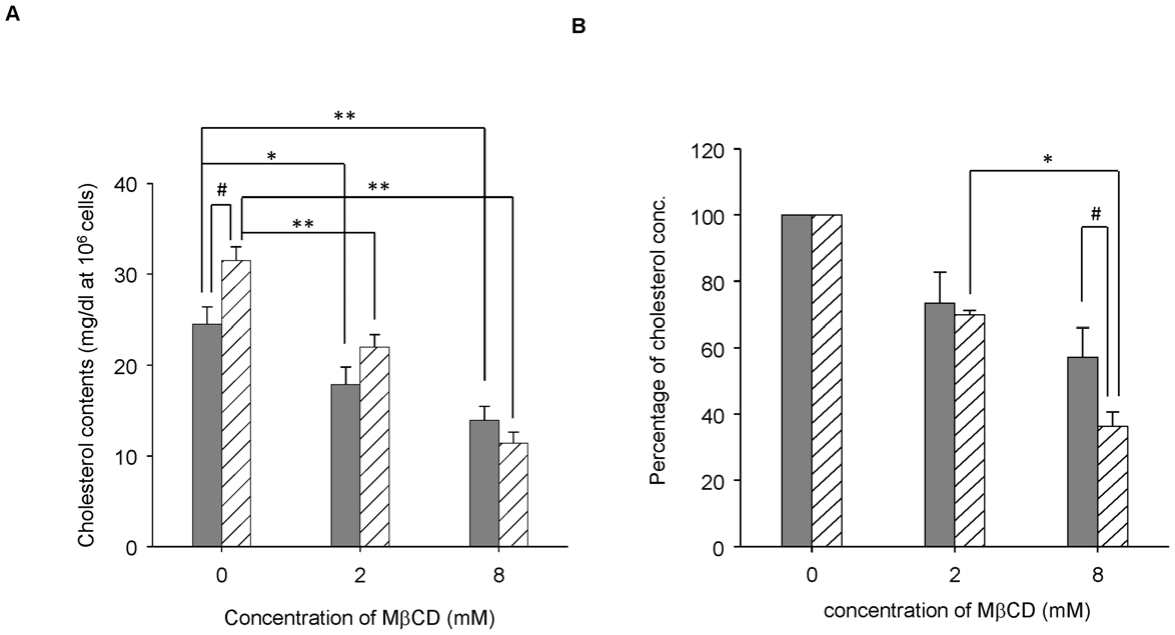
HeLa cells are highly susceptible to sequestration of cholesterol by MβCD. (A) Contents of cellular cholesterol were determined as described in “Materials and methods”. Gray bar indicates CHO cells, and shade bar indicates HeLa cells. *, *P*<0.05. **, *P*<0.01. #, *P*<0.01. (B) Percentage of cellular cholesterol was determined as described in “Materials and methods”. Gray bar indicate CHO cells, and shade bar indicate HeLa cells. *, *P*<0.01. #, *P*<0.05.

To compare the influence of MβCD between CHO cells and HeLa cells, we measured the cellular cholesterol contents and the percentage decrease of cholesterol in CHO and -HeLa cells treated with 0, 2, or 8 mM of MβCD. The cholesterol content (mg/1×10^6^ cells) in the HeLa cells without treatment with MβCD was significantly higher than in the CHO cells, but fell significantly in both cell lines to similar levels by treatment with 8 mM MβCD (Fig. 6A). However, if we compare the cholesterol reduction efficiency of MβCD between these two cell lines, the treatment with 8 mM MβCD was more effective in the HeLa cells than in the CHO cells, whereas the reduction efficiency at 2 mM MβCD was almost the same in both cell lines (Fig. 6B). These findings indicate that HeLa cells have a higher baseline cholesterol content, and have a higher susceptibility to MβCD than CHO cells.

### MβCD inhibits cytotoxicity by reducing VVH binding on HeLa cells

**Figure 7:**
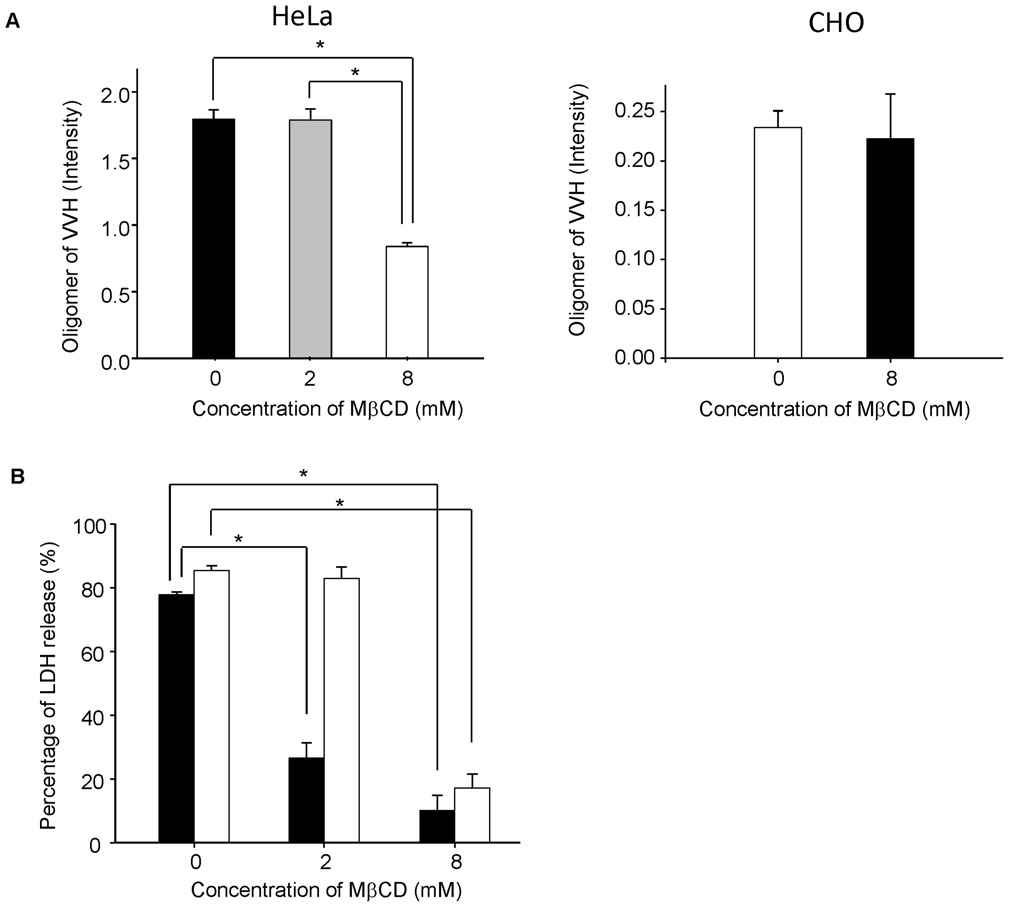
MβCD inhibits cytotoxicity by reducing VVH binding on HeLa cells. (A) Indicated concentrations of MβCD-untreated or -treated HeLa or CHO cells were incubated with 5 µg/ml of VVH for 15 min at 37°C and then the cells were lysed. The VVH oligomer was detected from cell lysate with anti-VVH polyclonal antibody by western blotting. The amount of oligomer was calculated as described in “Materials and methods”. *, *P*<0.01. (B) HeLa cells were incubated with indicated concentrations of MβCD or without at 37°C for 1 h. After incubation, the cells were incubated with 1 µg/ml of VVH (open bar) for 3 h or 50 HU/ml of SLO (closed bar) for 1.5 h at 37°C. The cytotoxicity in these cells was assayed by the release of LDH. Data are represented as the mean ± SD and represented three independent experiments, each in triplicate wells. *, *P*<0.01.

As shown in Fig. 5D, 8 mM MβCD inhibited the cytotoxicity of VVH in HeLa cells. To determine the inhibition mechanism of VVH cytotoxicity in these cells, we evaluated the binding efficiency and oligomer formation of VVH by measuring the amount of oligomer of VVH on HeLa cells and -CHO cells treated with 0, 2 or 8 mM of MβCD. The amount of oligomer decreased only in 8 mM MβCD-treated HeLa cells (Fig. 7A), and the monomer could not be detected in those cells (data not shown). On the other hand, the amount of oligomer did not decrease in 8 mM MβCD-treated CHO cells (Fig. 7A). It has previously been reported that VVH first binds to the cellular membrane as monomers and then these monomers are assembled to form oligomers [8]. Our results indicated that the amount of oligomer was decreased as a result of reduced monomer binding in 8 mM MβCD-treated HeLa cells.

Next, we confirmed whether or not the decrease of oligomer by the treatment of MβCD affected cytotoxicity of VVH in HeLa cells. Reflecting the results of HeLa cells in Fig. 7A, the percentage of LDH release by VVH decreased from 85.5±1.5% to 17.3%±4.3% in 8 mM MβCD-treated HeLa cells, whereas VVH cytotoxicity was not prevented in 2 mM MβCD-treated HeLa cells and in 8 mM MβCD-treated CHO cells (Fig. 7B). This demonstrates that 8 mM MβCD inhibits the cytotoxicity of VVH by decreasing the binding of VVH to HeLa cells. Remarkably, VVH induced cytotoxicity in 2 mM MβCD-treated HeLa cells, but SLO, a well known CDC, did not (Fig. 7B).

## Discussion

It was reported that several pore-forming toxins, such as aerolysin and TDH, can associate with DRM fractions, as determined by analysis using the sucrose gradient ultracentrifugation technique [20], [21], [22]. Aerolysin mainly associates with lipid rafts using a GPI-anchor protein as a receptor [28]. The localization of aerolysin was not affected by MβCD treatment [21], [22]. On the other hand, Matsuda et al. [20] reported that TDH localizes at DRM fractions and non-DRM fractions equally, and that all the detectable TDH was shifted to non-DRM fractions by sucrose gradient analysis in MβCD-treated HeLa cells. They also reported that TDH is shifted from DRM fractions to non-DRM fractions in SMase-treated HeLa cells [20], and concluded that TDH may localize at MβCD- and SMase-sensitive membrane regions (cholesterol and sphingomyelin rich membrane regions) [20]. In our experiments, the localization of VVH shifted from DRM fractions to non-DRM fractions in MβCD-treated CHO cells, but not in SMase-treated CHO cells, unlike aerolysin and TDH, respectively (Fig. 3). In addition, VVH did not co-localize on the cellular membrane with either of three lipid raft marker molecules or a control non-lipid raft marker by fluorescent microscope analysis (Fig. 4). These results indicated that VVH localized at membrane regions that were relatively abundant in cholesterol, and which were included in DRM fractions by sucrose gradient ultracentrifugation, but which were not identical with lipid rafts on cellular membrane. Thus, the localization of VVH on cellular membrane is possibly different from that of aerolysin or TDH.

The localization of VVH was drastically shifted from DRM fractions to non-DRM fractions by cholesterol sequestering with 8 mM MβCD in Fig. 2. Although the percentage of cellular cholesterol in CHO cells was decreased to 57.2±8.8% of the control by treatment with 8 mM MβCD (Fig. 6B), the total amount of VVH oligomer in these cells was not affected (Fig. 7A). Furthermore, there was no change in cytotoxicity of VVH between the MβCD-treated and the untreated cells at any concentration of VVH (data not shown). These findings demonstrate that cholesterol sequestering was able to influence the localization of VVH, but not binding efficiency, oligomer formation and cytotoxicity. Thus, it is clear that the most important determinant for exertion of cytotoxicity by VVH is the amount of oligomer on the cellular membranes.

It is well known that VVH binds to cellular membrane as a monomer and then forms oligomers by membrane fluidity [8]. As shown in Fig. 1, equal amounts of VVH monomers were detected from both DRM and non-DRM fractions. In addition, VVH has cytotoxicity against CHO cells, J774A.1 cells, Caco-2 cells, and HeLa cells (Fig. 5A, B, C and D). These data suggested that cellular receptors for VVH exist in both DRMs and non-DRMs equally in CHO cells, and that these receptors might be expressed in various cell lines. Cholesterol is thought to be a cellular receptor for VVH because its components are ubiquitously expressed on cellular membranes in mammalian cells. Certainly, the cytotoxicity of VVH on HeLa cells was prevented by 8 mM MβCD treatment (Fig. 7B). The amount of oligomer was also decreased in 8 mM MβCD-treated HeLa cells due to the decreased binding efficiency of VVH on these cells (Fig. 7A). However, when we compared the cholesterol contents between HeLa cells and CHO cells after 8 mM MβCD treating, there was no difference in the cholesterol contents in the two cell lines (Fig. 6A). These results suggest that cholesterol is not the main receptor for VVH cytotoxicity. In our experiment, the percentage of cellular cholesterol was decreased to 36.3±4.3% of the control in 8 mM MβCD-treated HeLa cells (Fig. 6B), and it is well known that cellular cholesterol is essential to maintain membrane stability, suggesting that such a severe decrease of cellular cholesterol by 8 mM MβCD might cause disruption of membrane stability in the HeLa cells. Therefore, a decrease in binding efficiency of VVH in 8 mM MβCD-treated HeLa cells might be the main mechanism for prevention of VVH cytotoxicity in the HeLa cells. Our conclusion that cholesterol is not the main cellular receptor for VVH is also supported by our experiment with SLO (Fig. 7B). The cytotoxicity of SLO was inhibited in 2 mM MβCD-treated HeLa cells but the cytotoxicity of VVH was not (Fig. 7B). These data also suggested that the cellular cholesterol might not be directly involved in the cytotoxic mechanism of VVH, and that VVH might not be a CDC.

Taken together, we showed that the localization of VVH on the cell membrane may be different from that of aerolysin and TDH. In the future, bacterial pore-forming toxins such as VVH, TDH and aerolysin may become useful tools for classification and/or tracking of specific regions on cellular membrane.

## Materials and Methods

### Cell culture

Chinese hamster ovary (CHO) cells (ATCC, number CCL-61), J774A.1 cells (ATCC, number TIB-67), Caco-2 cells (ATCC, number HTB-37) and HeLa cells (ATCC, number CCL-2.2) were grown in Dulbecco's modified Eagle's minimum essential medium (DMEM; Gibco BRL Life Technologies, Rockville, MD) supplemented with 2 mM glutamine, 2 mM sodium pyruvate, and 10% heat-treated fetal calf serum. Cells were incubated at 37°C under 5% CO2 in air in a humidified atmosphere.

### Reagents and antibodies

Methyl-β-cyclodextrin (MβCD), sphingomyelinase (SMase), streptolysin-O (SLO), biotin conjugated cholera toxin subunit B (CTxB) and Lysenin were purchased from Sigma (St. Louis, MO). Alexa 488-conjugated anti-rabbit anti-body, Alexa 546-conjugated anti-mouse anti-body and Alexa 546-conjugated streptavidin were purchased from Molecular Probes (Eugene, OR). The presence of actin, flotillin-1, transferrin receptor (TfR), caveoline-1 and VVH were detected using anti-actin monoclonal antibody clone C4 (Chemicon International Inc., Temecula, CA ), anti-flotillin-1 monoclonal antibody clone 18 (BD Biosciences, San Jose, CA), anti-TfR monoclonal antibody clone H68.4 (Zymed Laboratories Inc., South San Francisco, CA), anti-caveolin-1 monoclonal antibody clone 2297 (BD Biosciences, San Jose, CA) and anti-VVH polyclonal antibody, respectively.

### Purification of VVH

VVH was purified from the culture supernatant of the *V. vulnificus* K1 strain following the method of Oh et al. [29]. The protein concentration of each fraction was checked by optical density at 280 nm, and fractions with a high concentration of protein were used for sodium dodecyl sulfate polyacrylamide gel electrophoresis (SDS-PAGE). The gel was stained with staining solution containing 0.5% Coomassie brilliant blue R-250. Purified VVH was observed as a single band. VVH-containing fractions were dialyzed in 10 mM glycine buffer (pH 9.8)–150 mM NaCl at 4°C for 16 h. The dialyzed fractions were pooled as the purified VVH. The specific activity of purified VVH was 70,000 hemolytic units/mg (HU/mg), which was confirmed by examining the hemolytic activity against mouse erythrocytes.

### Isolation of DRMs

CHO cells were seeded in 8-cm tissue culture dishes at 2.5×10^6^ cells/dish. After 48 h, the cells were washed twice in DMEM and then incubated in medium containing 8 mM MβCD or 100 mU/ml SMase for 1 h at 37°C. The cells were rinsed and then incubated with 5 µg/ml VVH at 37°C for 15 min. To detect the monomer, cells were incubated for 1 h at 4°C with the same concentration of VVH. After incubation, the cells were lysed with 700 µl of lysis buffer (20 mM Tris-HCl, pH 7.4, 150 mM NaCl) supplemented with 1% Triton X-100 and a protease inhibitor mixture. Cells were scraped and left for 30 min on ice. After centrifugation at 800×g for 10 min, 500 µl of the postnuclear supernatant was mixed with 500 µl of 80% sucrose (wt/vol) in ice-cold lysis buffer without detergent and placed at the bottom of Hitachi 5PA ultracentrifuge tubes. The samples were overlaid with 2 ml of 30% sucrose and 1 ml of 5% sucrose and centrifuged (Hitachi koki, Tokyo, Japan) at 170,000×g for 18 h. Following centrifugation, eight fractions of 500 µl each were collected, starting at the top of the gradient. A distinct Triton X-100-insoluble whitish band that floated to the 5–30% interface was designated the DRMs fraction. The whole procedure was performed at 0–4°C. Aliquots of each fraction were boiled in SDS-PAGE sample buffer (0.01% bromophenol blue, 125 mM Tris-HCl, 20% glycerol, and 4% SDS) containing 16.5 mM dithiothreitol, loaded onto SDS-PAGE (10% polyacrylamide gel), and transferred to an Immobilon-P transfer membrane (Millipore Corporation, Bedford, MA) for 60 min at 15 V. Proteins on the blots were detected using specific antibodies and visualized using an enhanced chemiluminescence (ECL) system (Amersham Pharmacia Biotech, Piscataway, NJ) according to the manufacturer's protocol.

### Cytotoxicity assay

Cytotoxicity was determined using a lactate dehydrogenase (LDH) release assay. Cells were seeded in 24-well tissue-culture plates at 1×10^5^ cells/well. After 48 h, the cells were washed twice with DMEM, and then replaced with indicated concentrations of MβCD or indicated concentrations of SMase. After incubation with MβCD or SMase for 1 h at 37°C, the cells were washed twice with DMEM for VVH or PBS for SLO. The VVH or SLO was added and further incubated for 3 h or for 1.5 h at 37°C respectively. Then aliquots of medium samples (sample LDH) were assayed for LDH activity using pyruvate as a substrate. Cells treated with MβCD or SMase were used to assess background LDH activity. The percentage of LDH release was calculated as (sample LDH−background LDH)/(total LDH−background LDH)×100.

### Oligomerization assay

Cells were seeded in 6-well tissue-culture plates at 5×10^5^ cells/well. After 48 h, the cells were washed twice with DMEM, and then replaced with or without indicated concentrations of MβCD for 1 h at 37°C. The cells were incubated with 5 µg/ml of VVH for 15 min at 37°C and then extracted with lysis buffer supplemented with 1% Triton X-100 and a protease inhibitor mixture. VVH oligomer and cellular actin were detected by western blotting using antibodies against anti-VVH and anti-actin. The band intensity of these proteins was measured using NIH Image J software. Amount of oligomer was calculated by dividing the band intensity of oligomer by that of actin.

### Fluorescent microscope analysis

CHO cells were fixed with 3% (wt/vol) paraformaldehyde for 15 min. the cells incubated with 5 µg/ml VVH at 37°C for 15 min. After washing with PBS, the cells were incubated with anti-VVH and, anti-caveolin-1, anti-flotillin-1, biotin-conjugated cholera toxin subunit B (CTxB), or anti-TfR at room temp for 45 min. After washing with PBS, the cells were incubated with Alexa 488-conjugated anti-rabbit anti-body and Alexa 546-conjugated anti-mouse anti-body or Alexa 546-conjugated streptavidin at room temp for 45 min. Leica FW4000 microscope was used for fluorescent microscopy.

### Measurement of cholesterol contents

Cellular cholesterol contents were assayed spectrophotometrically using a Cholesterol E-Test Wako. (Wako, Osaka, Japan). Briefly, after treatment with MβCD, the cells were washed twice with 1 ml of cold PBS, and then lysed with lysis buffer. Six hundred fifty microliters of the cell lysate was mixed with 100 µl of the cholesterol assay kit buffer solution and then this mixture was further mixed with 750 µl of concentration enzyme mix solution. Samples were incubated for 5 min at 37°C prior to measuring absorbance at 600 nm. The cholesterol contents were determined as follows: (measured fluorescence of sample/fluorescence of standard cholesterol)×200. The percentage of remaining cholesterol after pretreatment with MβCD was determined as follows: (measured fluorescence of treated cells obtained from a standard curve/total fluorescence of untreated cells)×100.

### Statistical analysis

All results are shown as means ± SD (n = 3). For multiple comparisons of data, we used one-way ANOVA followed by Tukey's test; *P*<0.01 was considered statistically significant. Data from studies with only two groups were analyzed by Student's *t* test for equal variance or Welch's *t*-test for unequal variance after Bartlett's test.
